# Personal

**Published:** 1893-04

**Authors:** 


### Personal.

---

Dr. W. S. Renner has returned from his European trip.

---

Dr. Charles Cary is off for Europe for a few months' recreation.

---

Dr. Andrews, of the Buffalo State Hospital, is sojourning in California.

---

Dr. Cyrus Siegfried, of Allen street, has removed to 149 Franklin street.

---

Dr. Pettus, of the Marine Hospital staff, has been transferred to Southampton, Eng.

---

Passed-Assistant Surgeon Bratton, of Chicago, will succeed Dr. Pettus as Marine Hospital Surgeon in this city.

---

The many admirers of Dr. Robert Bartholow, of Philadelphia, will be pleased to learn of his ultimate recovery, and that he has again resumed his practice.

